# Pollinator richness, pollination networks, and diet adjustment along local and landscape gradients of resource diversity

**DOI:** 10.1002/eap.2634

**Published:** 2022-06-26

**Authors:** Carmelo Gómez‐Martínez, Miguel A. González‐Estévez, Joana Cursach, Amparo Lázaro

**Affiliations:** ^1^ Global Change Research Group Mediterranean Institute for Advanced Studies (UIB‐CSIC) Esporles Spain; ^2^ Department of Biology, Laboratory of Botany, Research Group on Plant Biology under Mediterranean Conditions University of the Balearic Islands Palma Spain; ^3^ Department of Biology, Ecology Area University of the Balearic Islands Palma Spain

**Keywords:** diet breadth, flower richness, functional complementarity, honey bees, landscape heterogeneity, modularity, neutral processes, pollinator abundance, specialization, wild pollinators

## Abstract

Loss of habitats and native species, introduction of invasive species, and changing climate regimes lead to the homogenization of landscapes and communities, affecting the availability of habitats and resources for economically important guilds, such as pollinators. Understanding how pollinators and their interactions vary along resource diversity gradients at different scales may help to determine their adaptability to the current diversity loss related to global change. We used data on 20 plant–pollinator communities along gradients of flower richness (local diversity) and landscape heterogeneity (landscape diversity) to understand how the diversity of resources at local and landscape scales affected (1) wild pollinator abundance and richness (accounting also for honey bee abundance), (2) the structure of plant–pollinator networks, (3) the proportion of actively selected interactions (those not occurring by neutral processes), and (4) pollinator diet breadth and species' specialization in networks. Wild pollinator abundance was higher overall in flower‐rich and heterogeneous habitats, while wild pollinator richness increased with flower richness (more strongly for beetles and wild bees) and decreased with honeybee abundance. Network specialization (*H*_2_′), modularity, and functional complementarity were all positively related to floral richness and landscape heterogeneity, indicating niche segregation as the diversity of resources increases at both scales. Flower richness also increased the proportion of actively selected interactions (especially for wild bees and flies), whereas landscape heterogeneity had a weak negative effect on this variable. Overall, network‐level metrics responded to larger landscape scales than pollinator‐level metrics did. Higher floral richness resulted in a wider taxonomic and functional diet for all the study guilds, while functional diet increased mainly for beetles. Despite this, specialization in networks (*d*′) increased with flower richness for all the study guilds, because pollinator species fed on a narrower subset of plants as communities became richer in species. Our study indicates that pollinators are able to adapt their diet to resource changes at local and landscape scales. However, resource homogenization might lead to poor and generalist pollinator communities, where functionally specialized interactions are lost. This study highlights the importance of including different scales to understand the effects of global change on pollination service through changes in resource diversity.

## INTRODUCTION

Wild pollinators are essential for plant reproduction and the maintenance of ecosystem function (Kremen et al., 2007; Ollerton et al., 2011). Literature over the last decades have described a global pollinator decline (Burkle et al., 2013; Potts et al., 2010); however, recent reviews suggest a less alarming situation (Guzman et al., 2021; Saunders et al., 2020), in which the decline mostly occurs in anthropogenic ecosystems (Herrera, 2019). Such decline is thought to be mainly driven by land‐use changes that lead to the homogenization of landscapes (Holzschuh et al., 2007) and communities (Gossner et al., 2016), affecting the availability of habitats and resources that pollinators need (Kremen et al., 2007). To predict and anticipate the effects of global change on the pollination service provided by wild pollinators, it is necessary to understand how variation in resource diversity affects pollinator communities and their pollination interactions.

**FIGURE 1:**
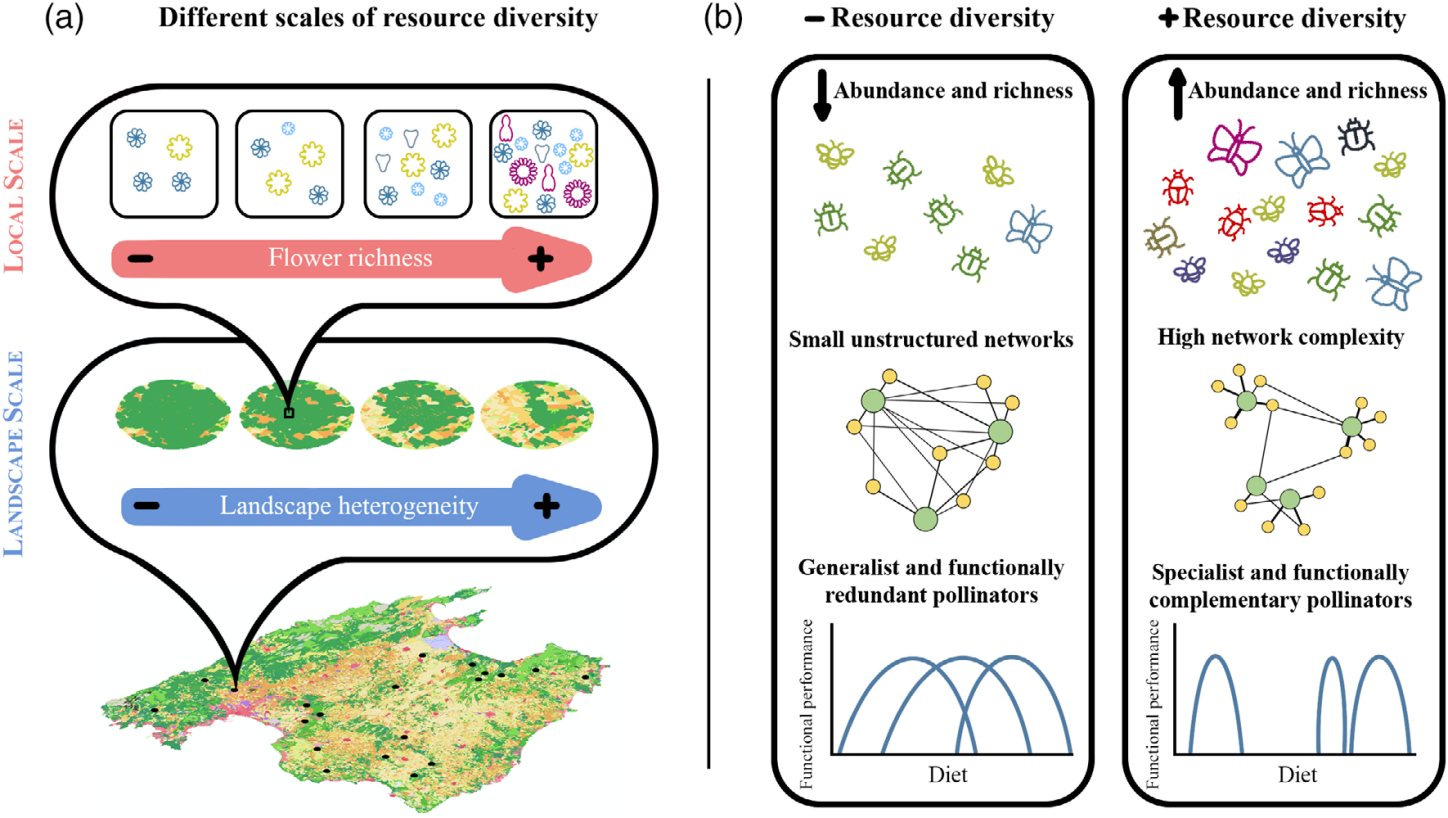
Conceptual diagram depicting the expected effects of resource diversity on pollinator communities and pollination interactions. (a) Variation in resource diversity at different scales. (b) Expected relationships between resource diversity and pollinator abundance and richness, the structure of plant–pollinator networks, and pollinators' diet breadth and specialization

Resource diversity varies both at landscape and local spatial scales (Figure 1a), affecting pollinators and their interactions differently (Moreira et al., 2015; Sjodin et al., 2008; Steffan‐Dewenter et al., 2002). At the landscape scale, an increase in the diversity of available habitats (landscape heterogeneity), may support more diverse pollinator communities (Figure 1b; Andersson et al., 2013, Mallinger et al., 2016, Ropars et al., 2020, Steckel et al., 2014), both because more habitats imply more niches to be exploited by different species (Holzschuh et al., 2008; Lázaro & Alomar, 2019) and because landscape heterogeneity might favor landscape complementation (i.e., the use of several habitats by pollinators to fulfill their needs for specific resources; Gathmann & Tscharntke, 2002, Klein et al., 2004). At the local scale, variation in flower diversity determines the availability of feeding resources (Potts et al., 2003), thus affecting the composition of pollinator communities (Figure 1b; Potts et al., 2003, Frund et al., 2010). However, different pollinators might have contrasting responses to resource variation at landscape or local scales. For instance, wild bees might be more sensitive than honeybees or hoverflies to variations in resource configuration (Blaauw & Isaacs, 2014), and different flying capabilities might influence pollinators' vulnerability to landscape modifications (Steffan‐Dewenter et al., 2002; Westphal et al., 2006). However, it is still unclear how and to what extent resource diversity at different scales affects the components of pollinator communities.

The structural properties of plant–pollinator networks may also change along gradients of resource diversity (Tylianakis & Morris, 2017). Previous work has shown increases in network specialization and modularity (Figure 1b) in relation to increases in landscape diversity (Escobedo‐Kenefic et al., 2020) and local flower diversity (Ebeling et al., 2011; Lázaro, Tscheulin, Devalez, Nakas, Stefanaki, et al., 2016; Traveset et al., 2018). Both metrics represent structural properties of ecological networks, typically linked to their stability (Gilarranz et al., 2017; Grilli et al., 2016; May, 1972). In addition, pollinator functional complementarity may also change along resource gradients (Figure 1b), because pollinators are expected to segregate niches to increase feeding efficiency and/or reduce competition (Blüthgen & Klein, 2011; Fründ et al., 2013; Venjakob et al., 2016), ultimately increasing network modularity and dietary specialization (Izquierdo‐Palma et al., 2021). Overall changes in network structure along gradients may be related to changes in species composition, but also to changes in interaction frequencies (Tylianakis & Morris, 2017). Pollination interactions are determined by trait matching and spatiotemporal constraints (Jordano et al., 2003; Santamaría & Rodríguez‐Gironés, 2007; Vázquez et al., 2009), as well as by neutral processes, i.e., abundant species interact more frequently and with more species than rare species if pollinators distribute themselves randomly across the plant community (Vázquez, 2005; Vázquez et al., 2009). It is likely that the relative importance of these processes vary along resource gradients, because pollinators are able to adjust their diet based on their preferences (Cusser et al., 2019; Kelly & Elle, 2021) and/or to reduce competition (Inouye, 1978; Jeavons et al., 2020; Pasquaretta et al., 2019; Wignall et al., 2020). Facilitation processes among plants for pollination (Braun & Lortie, 2019; Tur et al., 2016) might also modulate pollinators' perception of resource distribution and influence how they adjust their diets to improve foraging efficiency. It could be hypothesized that the greater range in resources in diverse communities might result in a higher frequency of actively selected interactions. However, to our knowledge, no study to date has directly assessed the influence of resource diversity at different scales on interaction patterns (random vs. actively selected interactions).

Diet adjustments along resource gradients may also influence wild pollinators' diet breadth (i.e., diversity of plant species and flower traits included in their diet; hereafter taxonomic and functional diet breath, respectively) and species' specialization (*d*′, Blüthgen et al., 2006) in the networks (Figure 1b3). Pollinators' taxonomic and functional diet breath might not be correlated in modified landscapes, because a high taxonomic diversity of plants is not necessarily associated with a high functional diversity (see, e.g., Cursach et al., 2020). Although species' specialization in the networks and diet breadth are expected to be negatively correlated, these variables may be unrelated if pollinators expand their diet but still make use of a small proportion of the available resources (Kelly & Elle, 2021). Similarly, specialization may be related or unrelated to functional complementarity, depending on whether specialist species share or not their functional niche (Blüthgen & Klein, 2011). Several studies, mostly in bees, have demonstrated that diet breadth and specialization (*d*′) change as a consequence of interspecific competition (Inouye, 1978, Jeavons et al., 2020, Pasquaretta et al., 2019, Wignall et al., 2020); however, only a few of them have assessed the influence of resource diversity on pollinators' diet (Cusser et al., 2019, Kelly & Elle, 2021). Understanding how pollinators' diets vary across gradients of resource availability may allow us to address community functions more accurately (Kelly & Elle, 2021).

In this study, we evaluate how resource diversity at landscape (landscape heterogeneity) and local (flower richness) scales affect wild pollinator richness and abundance, the structure of pollination networks and the use of flowering resources. For this, we used data on 20 natural plant–pollinator communities along gradients of landscape heterogeneity and flower richness. As the abundance of managed honeybees is known to affect wild pollinator diversity and abundance (Lázaro et al., 2021; Magrach et al., 2017; Ropars et al., 2019), we also assessed the effect of honeybee densities when testing the effects of resource diversity. Particularly, we aimed at evaluating the following hypotheses: (1) Wild pollinator abundance and richness will increase with resource diversity at both spatial scales, but at different rates for different pollinator guilds due to their different life history traits; (2) A high abundance of honeybees will negatively affect wild pollinator abundance and richness; (3) Pollination networks will become more complex in richer plant communities within heterogeneous landscapes; (4) The proportion of plant species that will be selected more than expected at random (i.e., those interactions not occurring by neutral processes) will change with local flower richness and changes in pollinator composition related to landscape heterogeneity; And (5) local flower diversity will increase wild pollinators' diet breadth, but also their species' specialization in networks, as pollinators visit a smaller subset of available plants.

## METHODS

### Study sites

This study was conducted on 20 wild *Olea europea* communities (study sites, hereafter) across Mallorca (39°37′ N, 2°59′ E), the main island of the Balearic Archipelago (Spain) in the Western Mediterranean Basin. For further information about the study area, see Cursach et al. (2020). We chose these communities because they contain a high diversity of species and can be found within diverse landscape compositions and configurations across the island (see Appendix S1: Table S1 in for geolocation of study sites). We selected study sites ≥1 ha, separated at least by 2 km (average distance between closest pairs: 6.5 ± 3 km), and located within agricultural landscapes, far away from the sea and urban areas.

### Diversity at the landscape scale

To describe the characteristics of the landscape surrounding the 20 1‐ha study sites, we established buffer zones with radii of 250 m, 500 m, and 1 km around the center of each study site, and calculated the area covered by different land‐cover layers using the CORINE database (EEA, 2018) and the software ArcMap v. 10.5 (ESRI, 2016). We did not use radii of more than 1 km, because previous studies showed that most of the pollinators have small foraging ranges (not larger than 1‐km radius, e.g., Osborne et al., 2008; Walther‐Hellwig & Frankl, 2000; Zurbuchen et al., 2010). In total, we recorded 19 land‐cover layers, including different natural and seminatural habitats (mainly conifer, mixed and hardwood forests, transition woodlands, scrubland, rocky zones, pastureland), crops (mainly tree cultivars as almond and carob trees) and some small artificial infrastructures/constructions.

As a measure of resource diversity at landscape scale, we used landscape heterogeneity, calculated as the Shannon's diversity index (Shannon, 1997) of all the cover layers in 250 m, 500 m, and 1 km buffer zones, using the R package *vegan* v. 2.5‐5 (Appendix S1: Table S1; Oksanen et al., 2019). We included all the cover layers in these analyses, because urban/suburban areas (Baldock et al., 2015, 2019; Hall et al., 2017) and extensively managed crops (Brosi et al., 2008; Winfree et al., 2007) are often favorable habitats for pollinators.

### Diversity at the local scale

We established a total of 30 1 × 1 m squares at each study site, 10 along each of the three permanent transects used to observe pollinators (see below the *Pollinator interactions* section), and separated by 10 m. The number of open flowers of each plant species within the squares was counted each day that pollinators were sampled (see below) at each study site.

To describe diversity at the local scale, we calculated flower richness as the total number of flowering plant species recorded across all the squares and sampling days at each study site (Appendix S1: Table S1). We chose flower richness instead of the Shannon's diversity of flowers, because these two variables were highly correlated (*r* = 0.70; *t* = 4.12, df = 18, *p* = 0.001), and the simplest measurement (flower richness) was the variable that better explained the observed patterns (models with lower AIC_c_ values).

### Pollinator interactions

To record foraging insects at each of the 20 wild *Olea europea* study communities, we established three permanent transects of 100 × 2 m within each study hectare, two in the extremes and one in the center. In total, we visited each study site seven different days in 2018: 5 days during the spring months (March–June) and 2 days in autumn, as this second flowering peak was shorter than the first one (from October to beginning of November), periods that corresponded to the main flowering peaks in these communities. Within each flowering peak, samplings at the same site took place every second week, between 10:00 and 16:00, on sunny days that favored pollinators' activity. Insects were recorded as we walked slowly along the three permanent transects for 1 h, noting each pollinator that was observed contacting the reproductive parts of a flowering plant. Additional information regarding sampling completeness of pollinators, plants, and interactions is shown in Appendix S1: Table S2. When the identification of insects to the species level was not possible in the field, we collected them for further identification in the lab, stopping the watch during insect manipulations to standardize the time spent per study site. Collected specimens were deposited at the pollinator collection of the Mediterranean Institute for Advanced Studies (UIB‐CSIC).

With these data we calculated the following: (1) Wild pollinator abundance in total and by guild (beetles, butterflies, flies, wasps, and wild bees), as the number of individuals of wild species (all the species registered except *Apis mellifera*) recorded in each study site. Hoverflies and bee flies were combined with other flies due to their low number. (2) Honeybee abundance, as the total number of individuals of *Apis mellifera* registered in a study site. And (3) wild pollinator richness in total and by guild, as the number of wild pollinator species recorded in each study site. Although sampling effort at each study site was similar, sampling completeness of wild pollinator species varied among sites (Appendix S1: Table S2). To account for the effect of sampling completeness on wild pollinator richness, we carried out an estimation of wild pollinator richness by applying the interpolation–extrapolation method of Chao et al. (2014), using rarefied estimates at 50% sampling completeness, which was the percentage of sampling completeness at the least completed site (Appendix S1: Table S2). For that, we used the function iNEXT from the R package *iNEXT* v. 2.0.20 (Hsieh et al., 2016). Pollinator abundance and raw and standardized pollinator richness for each study site are shown in Appendix S1: Table S3. To calculate the standardized richness per pollinator guild, we randomly subsampled species from the database to equal the overall standardized pollinator richness at each study site (Gotelli & Colwell, 2001). We repeated the procedure 1000 times for each study site and used the average number of species per guild as the estimate of standardized pollinator richness per guild (Appendix S1: Table S4 for raw and standardized pollinator richness per pollinator guild).

### Overall structure of pollination networks

We built 20 quantitative plant–pollinator interaction matrices, one per study site, using the total number of visits by each pollinator species to each plant species as link weight (seven sampling rounds pooled). Then, we used the R package *bipartite* v. 2.14 (Dormann et al., 2008) to calculate the following weighted network‐level metrics: (1) Network specialization (*H*_2_′), that measures the overall level of specialization of the interacting species in a network (Blüthgen et al., 2006). It varies from 0 (fully generalized network) to 1 (fully specialized network) and is calculated using the function *networklevel*. (2) Modularity, using function *metaComputeModules*, which describes the extent to which the networks are organized into subsets composed by strongly interlinked species weakly connected to other subsets (e.g., Dormann & Strauss, 2014; Olesen et al., 2007). And (3) functional complementarity, as pollinators' niche differentiation regarding the plant species they visit (function *grouplevel*, Blüthgen & Klein, 2011, Devoto et al., 2012). Networks used to calculate these metrics included both wild pollinators and honeybees; however, excluding honeybees from the networks did not change the results (see Appendix S1: Table S5). Correlations among these study network metrics and other metrics typically calculated in literature are shown in Appendix S1: Table S6).

Although time devoted to each study site was similar, networks of different sites varied in size. To obtain standardized matrices that could be comparable along the gradient, we calculated *z* scores for each study network metric (Izquierdo‐Palma et al., 2021; Lázaro et al., 2021; Vanbergen et al., 2017). For this, we first created 1000 null models using the *vaznull* function (Vázquez et al., 2007). These null models maintain connectance, number of interactions, and plant and pollinator richness, while randomizing individual interactions within the network. We then calculated *z* scores as [*x* − μ]/σ, with *x* being the observed metric value, μ the mean, and σ the standard deviation of the metric value for the 1000 null models. Negative *z* scores mean that the observed metric is lower than the expected at random distribution of interactions, while positive *z* scores mean that observed values are higher than these expected at random.

### Neutral processes versus active selection of flowers as determinants of interaction patterns

To evaluate how interaction patterns vary with local flower richness and landscape heterogeneity, we calculated, for each species of beetle, fly, and wild bee in the network, the proportion of its interactions with flowering plant species that occurred more than expected based on the relative abundance of the plant species (hereafter actively selected interactions), using R‐package *econullnetr* v. 0.2.0 (Vaughan et al., 2018). We did not analyze the proportion of interactions that occurred less than expected by plant abundance (avoided interactions; 4.6% ± 0.42% of total interactions [mean ± SE]) because, in networks constructed by pooling different sampling rounds, this value may include phenologically forbidden links. The proportion of actively selected interactions describes the relative importance of different processes underlying the observed plant–pollinator interactions (random selection vs. preferential selection of plant species). In this analysis, we only included beetles, flies, and wild bees because the rest of the groups were found in low numbers. This variable was not significantly correlated to functional complementarity or *d*′ for most groups (Appendix S1: Figure S1).

### Diet breadth and species' specialization in networks

To study how local flower richness and landscape heterogeneity influenced pollinator diet breadth and specialization for beetles, flies, and wild bees, we calculated (1) taxonomic diet breadth, as the total number of plant species visited by a pollinator, calculated as the species degree (function *specieslevel* in R package *bipartite* v. 2.14; Dormann et al., 2008); (2) functional diet breadth, as the functional dispersion (function *fdisp* in R package *FD* v. 1.0.12; Laliberté & Legendre, 2010) of the plant traits (floral unit, floral unit size, floral symmetry, flower color, flowering onset, and flowering length) included in pollinators' diet (see Appendix S1: Table S7 for details on the traits and sources of information); and (3) specialization (*d*′), which measures the extent of specialization of a pollinator species based on its interaction frequencies and the interaction frequencies of the rest of pollinator species in the network (Blüthgen et al., 2006), and is calculated with the function *specieslevel* in R package *bipartite* v. 2.14 (Dormann et al., 2008). It varies from 0 (completely generalist; when the pollinator visits a large number of species also visited by other species) to 1 (extremely specialist; when the pollinator visits a narrow subset of species not visited by other pollinators). To avoid sampling biases in the analyses of diet breadth, we only included in these calculations those pollinator species that (1) were present in at least 10 study sites, to have a good representation of the species along the diversity gradient, and (2) for which we had recorded at least five visits per study site, to be able to detect differences in their diet (for information about the species included in the analyses see Appendix S1: Table S8).

### Statistical analysis

All statistical analyses presented here were conducted in the software R v.4.0.2 (R Core Team, 2018). To evaluate how local flower richness and landscape heterogeneity affected overall wild pollinator abundance and richness and the structure of plant–pollinator networks, we performed generalized linear models (GLMs) for each response variable (wild pollinator abundance, wild pollinator richness, *H*_2_′, modularity, and functional complementarity). These models included flower richness and landscape heterogeneity as predictor variables, plus honeybee abundance in the models of overall wild pollinator abundance and richness, after checking for the absence of collinearity (VIF values <3; Zuur et al., 2009). We used a negative binomial distribution with log link function (function *glmer.nb* from the R package *MASS* v.7.3.51.4, Venables & Ripley, 2002) for the models of overall wild pollinator richness and abundance due to overdispersion (Zuur et al., 2009) and Gaussian distribution with identity link function for the models of H_2_′, modularity, and functional complementarity (function *glm* from the R package *stats* v.3.6.2, R Core Team, 2018) because they met the assumptions of normality (tested using the function *lillie.test* in R package *nortest* v.1.0.4, Gross & Ligges, 2015). As the values of landscape heterogeneity at different scales (250, 500 m, and 1 km radii) were collinear and could not be included together as predictive variables in the same model, we built three separate models, each one including the landscape heterogeneity at a different scale. Flower richness and honeybee abundance were not correlated with any of the landscape heterogeneity variables (Appendix S1: Figure S2). We performed automatic model selection based on AIC_c_ (function *dredge* in R package *MuMIn*, Barton, 2020) to select the most parsimonious model in each case, limiting the maximum number of predictive variables to two to avoid over‐parametrization (Harrell et al., 1984; Peduzzi et al., 1996). We also used AIC_c_ to select the most parsimonious model among the three best models at different landscape heterogeneity scales. We present the best models in the text and alternative models (ΔAIC_c_ ≤ 2), if any, in the Appendix S1: Table S9.

We used generalized linear mixed models (GLMMs, *lme4* v.1.1.23, Bates et al. 2015) to evaluate whether flower richness and landscape heterogeneity had contrasting effects on the abundance and richness of different pollinator guilds, and on the proportion of actively selected interactions. In these GLMMs, we added the interaction between pollinator guild and resource diversity variables and included the study site as random effect. As Poisson models for abundance by guild were overdispersed (Zuur et al., 2009), we used a negative binomial distribution with log link function (function *glmer.nb*) for them; Poisson distribution with log link function was used for the model of wild pollinator richness by guild (function *glmer*) after checking for the absence of overdispersion (Zuur et al., 2009); and binomial distribution with link logit was used to study the proportion of actively selected interactions (function *glmer*). We also used GLMMs to assess how local flower richness and landscape heterogeneity influenced wild pollinators' taxonomic and functional diet breadth and species' specialization in networks. Same as previous models, these GLMMs included local flower richness, landscape heterogeneity, and pollinator guild and its interactions with the other variables, but in this case, both the study site and the pollinator species were included as crossed random effects to account for pseudoreplication. We used Poisson distribution (link log) for the models of taxonomic diet breadth and gamma distribution (link log) for the models of functional diet breadth and species' specialization in networks (*d*′). As for the GLMs, we again built three models for each response variable, each one including landscape heterogeneity at a different scale, and then selected the best one with the lowest AIC_c_. For all the GLMMs, when an interaction was significant, we performed post‐hoc test to study the differences between guilds in their response to resource diversity in both means and trends (functions *lsmeans* and *lstrends* from the R package *emmeans* v.1.4.8, Lenth, 2020). Means are accompanied by their standard error throughout the text.

## RESULTS

We recorded 167 flowering plant species in our communities (42.95 ± 2.45 species per study site), and for 108 of these plant species (18.95 ± 2.06 per study site) we recorded 9635 visits of pollinators to flowers: 6983 by beetles (24 families, 78 species), 1136 by wild bees (five families, 67 species), 1276 by flies (33 families, 125 species), 142 by wasps (17 families, 44 species), 98 by butterflies (eight families, 24 species), and 1370 by honeybees. Total wild pollinator abundance varied from 224 to 868 individuals across the 20 study sites with a mean of 481.75 ± 44.01 individuals per site (Appendix S1: Table S3). Raw wild pollinator richness varied from 40 to 88, with a mean of 61.85 ± 3.38 species per site, while standardized wild pollinator richness (hereafter Wild pollinator richness), ranged from 34 to 67, with a mean of 47.95 ± 2.37 species per site (Appendix S1: Table S3). Honeybees were present in all the study sites, but their abundance was relatively low, and observed visitation events ranged from 6 to 140 across the 20 study sites, with a mean of 68.50 ± 10.78 honeybee visits to flowers per site (Appendix S1: Table S3). Information about richness of the different guilds can be found in Appendix S1: Table S4.

### Wild pollinator abundance and richness

**FIGURE 2:**
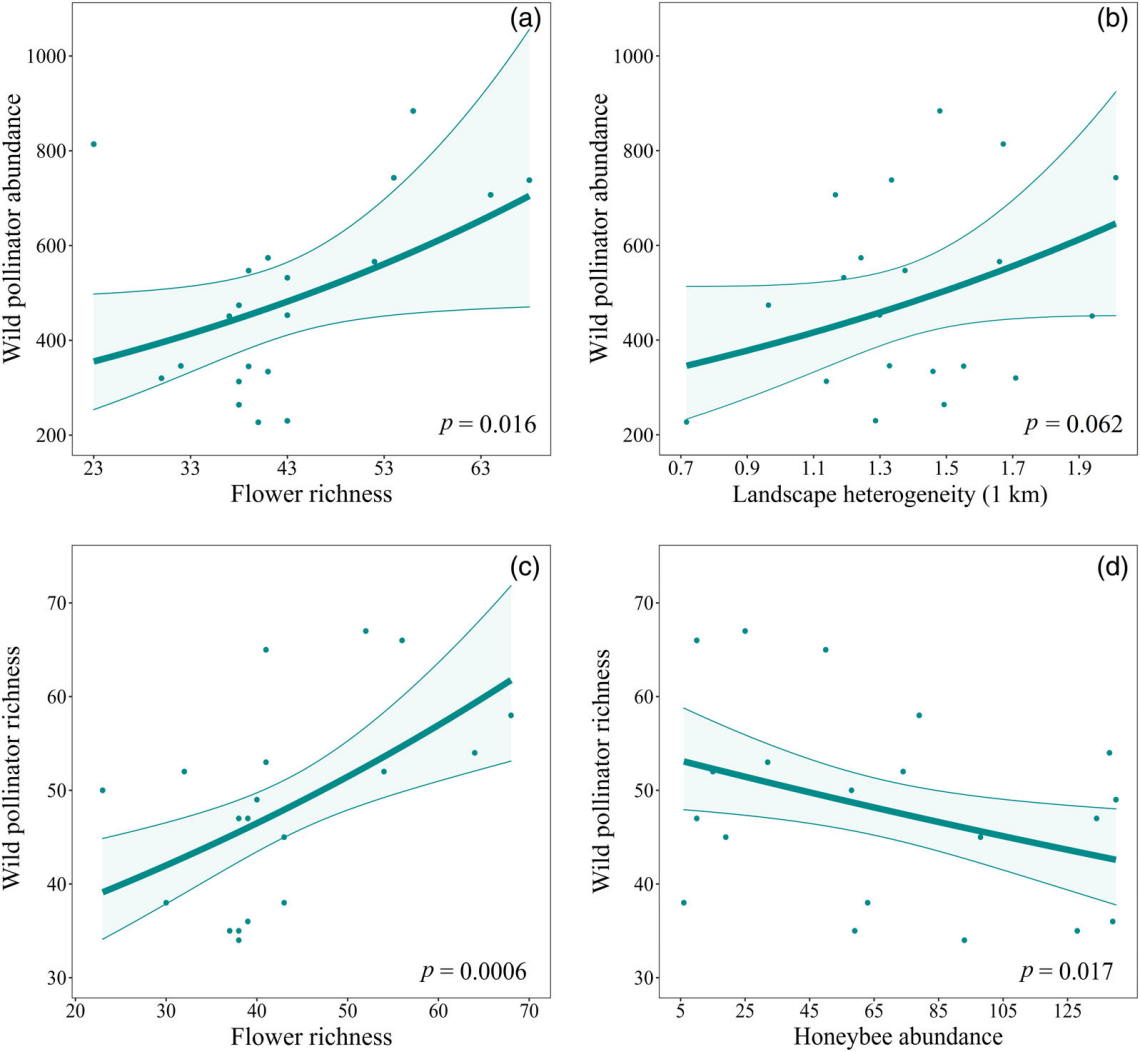
Effects of local flower richness, landscape heterogeneity, and honeybee abundance on wild pollinator abundance and richness. Plots show the relationships between wild pollinator abundance and (a) local flower richness and (b) landscape heterogeneity and between standardized wild pollinator richness and (c) local flower richness and (d) honeybee abundance. The lines represent the estimates of the best models, the dots represent the data for each study site, and the shaded area the confidence interval

The best model of total wild pollinator abundance showed an increase in the number of pollinators with both flower richness and landscape heterogeneity at 1 km, although the last relationship was only marginally significant (Table 1a and Figure 2a,b). Alternative models to this best one (∆AIC_c_ ≤ 2) contained separately only flower richness or landscape heterogeneity as predictor variables (Appendix S1: Table S9). When pollinator abundance was analyzed by guilds, we found clear significant differences in abundance among the guilds (Table 1b), beetles being the most abundant guild (349.15 ± 37.22 individuals per site), followed by wild bees (56.8 ± 8.92), flies (63.8 ± 6.20), wasps (7.1 ± 0.83), and butterflies (4.9 ± 1.15). However, we did not detect any significant interaction between the pollinator guild and resource diversity at local or landscape scales (Table 1b).

**TABLE 1:** Results of the best models showing the relationships between flower richness and landscape heterogeneity and (a) total wild pollinator abundance, (b) wild pollinator abundance by guild, (c) total wild pollinator richness, and (d) wild pollinator richness by guild

Predictor	*χ* ^2^	df	*p*
Model			
(a) Overall wild pollinator abundance			
Flower richness	5.81	1	**0.016**
Landscape heterogeneity (1 km)	3.48	1	0.062
(b) Wild pollinator abundance by guild			
Flower richness	3.22	1	0.073
Landscape heterogeneity (1 km)	2.37	1	0.123
Pollinator guild	25.48	4	**<0.0001**
Flower richness × Pollinator guild	6.495	4	0.165
Landscape heterogeneity (1 km) × Pollinator guild	2.76	4	0.600
(c) Overall wild pollinator richness			
Flower richness	11.73	1	**0.0006**
Honeybee abundance	5.68	1	**0.017**
(d) Wild pollinator richness by guild			
Flower richness	6.55	1	**0.010**
Pollinator guild	34.51	4	**<0.0001**
Honeybee abundance	6.32	1	**0.012**
Flower richness × Pollinator guild	11.62	4	**0.020**

*Notes*: The χ^2^, the degrees of freedom (df), and the *p* values were calculated based on likelihood ratio tests. Significant *p* values are shown in boldface type.

**FIGURE 3:**
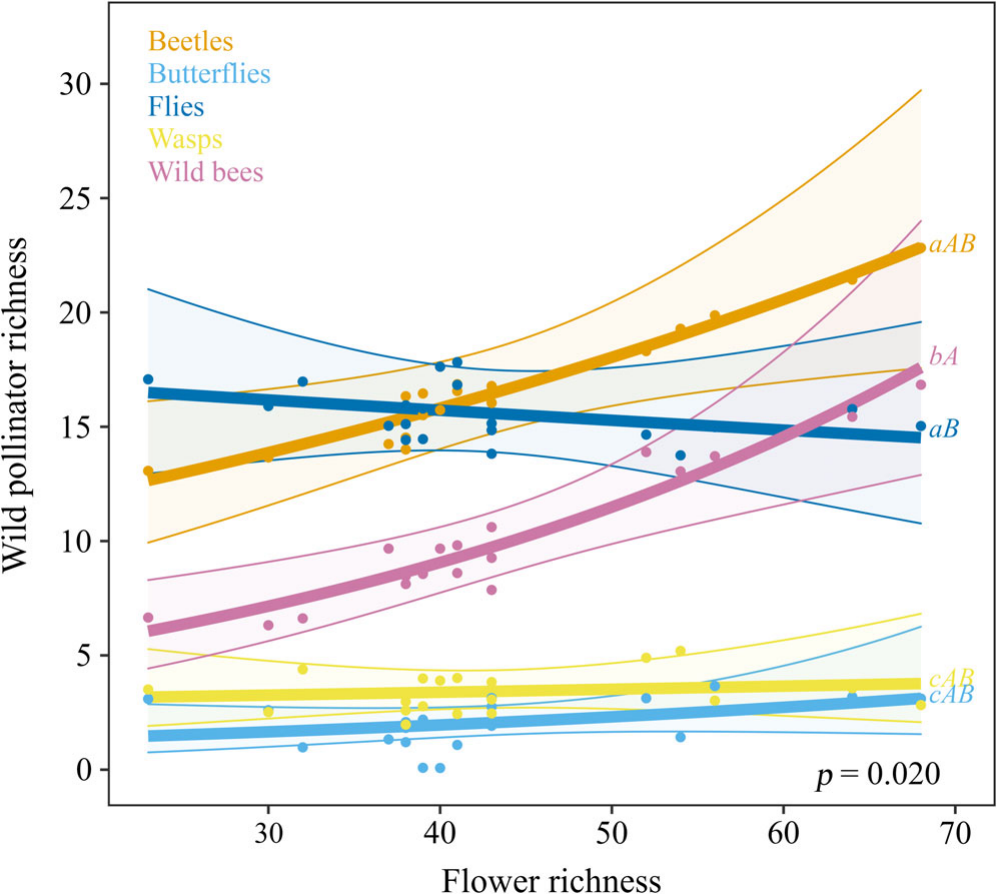
Effect of local flower richness on wild pollinator richness by guild. The plot shows the relationship between local flower richness and the standardized richness of the different wild pollinator guilds. The lines represent the estimates of the best model for each pollinator guild, the dots represent the data for each pollinator guild at each study site, and the shaded area the confident interval. Whenever there was an interaction with pollinator guild, different letters indicate significant differences in mean (estimated marginal means, lowercase) and trends (estimated marginal means of linear trends, uppercase)

The best model of total wild pollinator richness showed that the number of pollinator species also increased with flower richness, while it decreased with the abundance of honeybees in the communities (Table 1c and Figure 2c,d). The analysis of wild pollinator richness by guild indicated that the increase in overall wild pollinator richness with flower richness was mainly driven by the increase in beetle and wild bee richness (Table 1d and Figure 3). However, a posteriori analysis of trends indicated that only wild bees and flies differed significantly in their rate of change in response to flower richness: while wild bee richness increased fast with flower richness, fly richness remained constant along the whole flower gradient (Figure 3). Overall, beetles were the richest guild (16.7 ± 0.93 species per site), followed by flies (15.7 ± 1.08), wild bees (10.15 ± 0.93), wasps (3.45 ± 0.38), and butterflies (2.10 ± 0.33). These results did not change when dipterans were separated in bee flies, hoverflies, and other flies (Appendix S1: Figure S3 and Table S11).

### Structure of pollination networks

**FIGURE 4:**
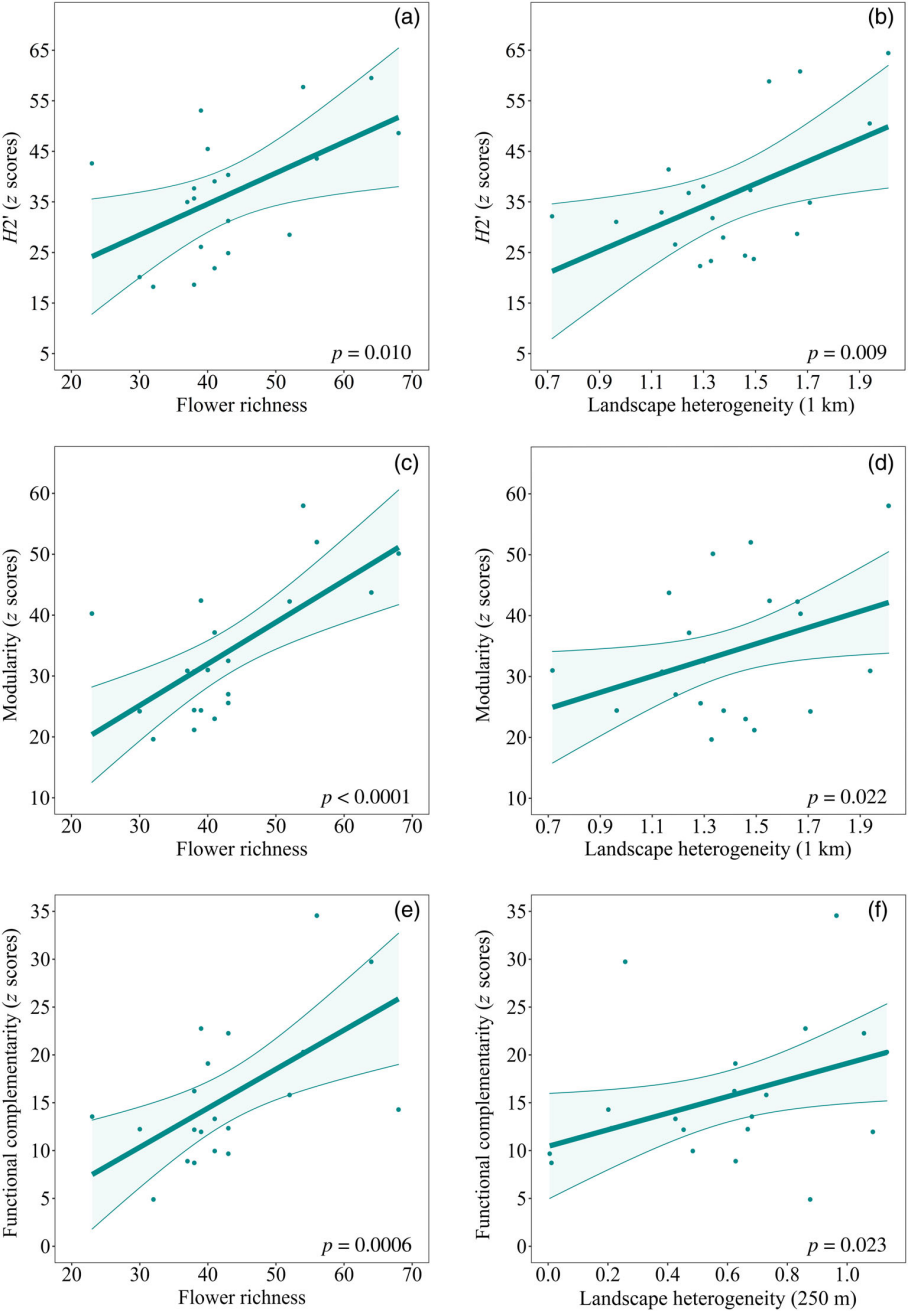
Effect of local flower richness and landscape heterogeneity on network structure. Plots showing the relationships between resource diversity at different scales (flower richness and landscape heterogeneity) and *z* scores of (a, b) network specialization (*H*_2_′), (c, d) modularity, and (e, f) functional complementarity. The lines represent the estimates of the best models, the dots represent the *z* scores for each study site, and the shaded area the confidence interval

The *z* scores of network specialization (*H*_2_′), modularity, and functional complementarity were all positive and significant (Appendix S1: Table S10), indicating higher values than those expected at random. All, *H*_2_′, modularity, and functional complementarity increased with both flower richness and landscape heterogeneity (Table 2a–c and Figure 4a,b). While *H*_2_′ and modularity responded to landscape heterogeneity at 1 km, functional complementarity responded to the landscape at a smaller scale of 250 m (Table 2a–c and Figure 4a,b).

**TABLE 2:** Results of the GLMs showing the relationships between flower richness and landscape heterogeneity and *z* score values of (a) network specialization (*H*_2_′), (b) modularity, and (c) functional complementarity

Predictor	*χ* ^2^	df	*p*
Model			
(a) Network specialization (*H*_2_′)			
Flower richness	6.58	1	**0.010**
Landscape heterogeneity (1 km)	6.84	1	**0.009**
(b) Modularity			
Flower richness	17.41	1	**<0.0001**
Landscape heterogeneity (1 km)	5.22	1	**0.022**
(c) Functional complementarity			
Flower richness	11.67	1	**0.0006**
Landscape heterogeneity (250 m)	5.13	1	**0.023**

*Notes*: The χ^2^, the degrees of freedom (df), and the *p* values were calculated based on likelihood ratio tests. Significant *p* values are shown in boldface type.

### Neutral processes versus active selection of flowers as determinants of interaction patterns

**FIGURE 5:**
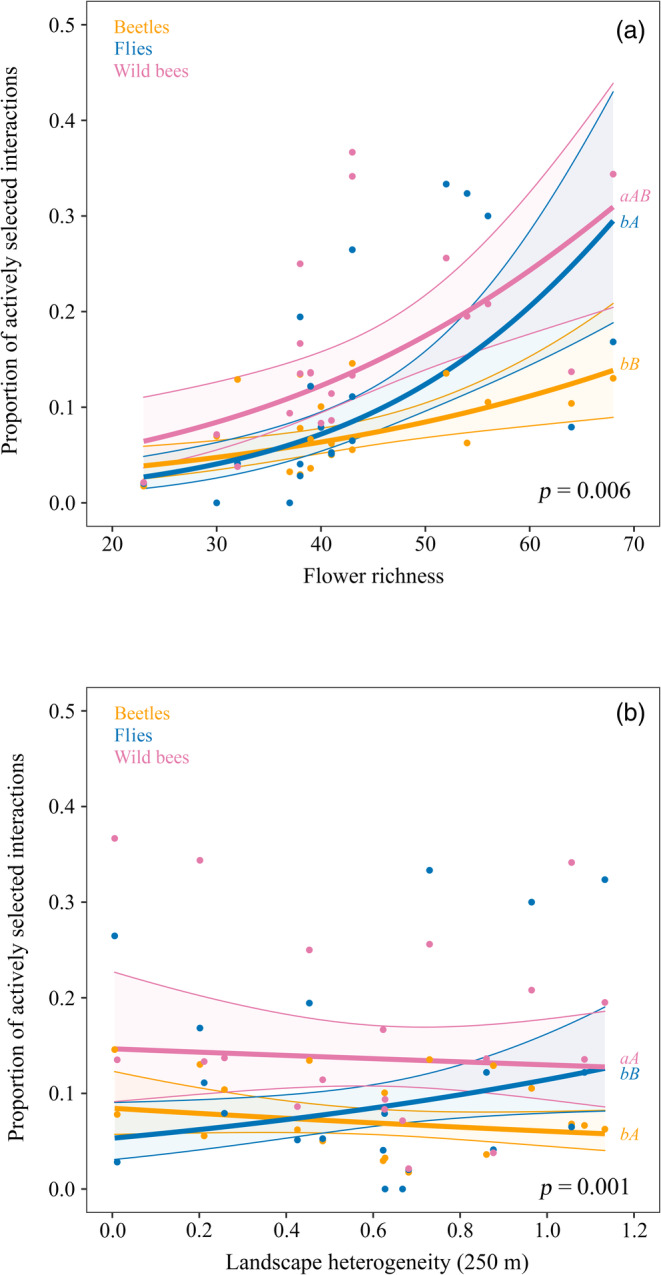
Effects of local flower richness and landscape heterogeneity on the proportion of actively selected interactions. Plots showing the relationships between the proportion of actively selected interactions (i.e., those that occur more often than expected at random) and (a) local flower richness and (b) landscape heterogeneity. The lines represent the estimates of the best model for each pollinator guild, the dots represent the data for each pollinator guild at each study site and the shaded area the confidence interval. Whenever there was an interaction with pollinator guild, different letters indicate significant differences in mean (estimated marginal means, lowercase) and trends (estimated marginal means of linear trends, uppercase)

Overall, pollinator guilds differed in their proportion of actively selected interactions (Table 3 and Figure 5), being wild bees involved in a significantly higher proportion of actively selected interactions (0.17 ± 0.02), than flies (0.12 ± 0.02), and beetles (0.08 ± 0.01). The proportion of actively selected interactions increased with flower richness for the three study guilds, however the effect was significantly stronger for flies than for beetles (Table 3 and Figure 5a). The effect of landscape heterogeneity on the proportion of actively selected interactions also differed significantly between guilds (Table 3). Beetles and wild bees showed a very slight decrease in their proportion of actively selected interactions with landscape heterogeneity, while flies increased it (Figure 5b).

**TABLE 3:** Results of the generalized linear mixed models (GLMM) showing the relationship between resource diversity at different scales and the proportion of actively selected interactions (proportion of interactions occurring more than expected by the relative abundance of the flowering plant species).

Predictor	*χ* ^2^	df	*p*
Flower richness	10.31	1	**0.001**
Pollinator guild	10.49	2	**0.004**
Landscape heterogeneity (250 m)	1.36	1	0.244
Flower richness × Pollinator guild	10.38	2	**0.006**
Landscape heterogeneity (250 m) × Pollinator guild	13.77	2	**0.001**

*Notes*: The χ^2^, the degrees of freedom (df), and the *p* values are calculated based on likelihood ratio tests. Significant *p* values are shown in boldface type.

### Diet breadth and species' specialization in networks

**FIGURE 6:**
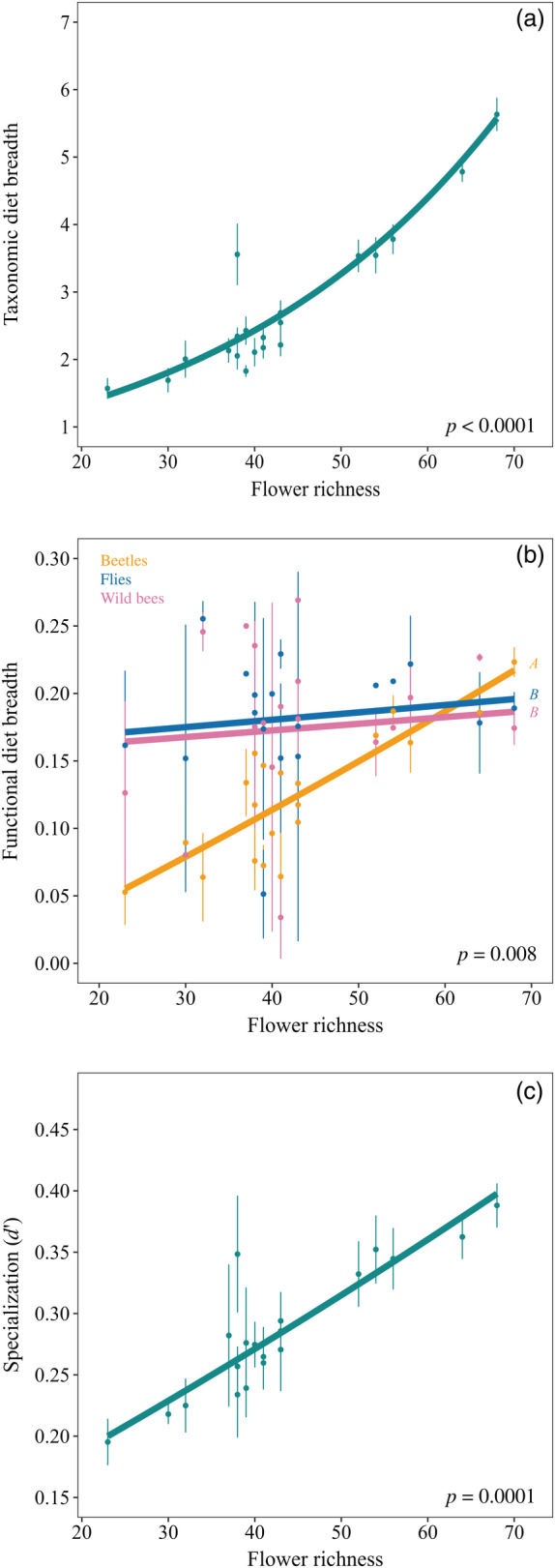
Effect of local flower richness on wild pollinators' diet breadth and specialization in networks (*d*′). Plots showing the relationships between local flower richness and (a) taxonomic diet breadth, (b) functional diet breadth, and (c) specialization in networks (*d*′). The lines represent the estimates of the best models, the dots represent the partial residuals and the vertical lines, standard errors. In (b), there was no significant differences in mean (estimated marginal means), while significant differences in trends (estimated marginal means of linear trends) are indicated by uppercase letters.

Taxonomic diet breadth increased with flower richness consistently for all the three main guilds (Table 4a and Figure 6a). However, functional diet breadth increased with flower richness, but differently for the three study guilds (Table 4b and Figure 6b): while beetles rapidly and sharply increased their functional diet breadth with flower richness, the functional diet breath of flies and wild bees remained relatively constant along the gradient (Table 4b and Figure 6b). Lastly, species specialization (*d*′) also increased with flower richness, consistently for all the study guilds (Table 4c and Figure 6c).

**TABLE 4:** Results of generalized linear mixed models (GLMMs) showing the relationships between landscape heterogeneity and local flower richness and wild pollinators' (a) taxonomic diet breadth (number of plant species visited by pollinators), (b) functional diet breadth (functional dispersion, Laliberté & Legendre, 2010), and (c) specialization (*d*′, Blüthgen et al., 2006)

Predictor	*χ* ^*2*^	df	*p*
Model			
(a) Taxonomic diet breadth			
Flower richness	78.85	1	**<0.0001**
Landscape heterogeneity (250 m)	2.75	1	0.097
(b) Functional diet breadth			
Flower richness	11.76	1	**0.0006**
Pollinator guild	12.99	2	**0.001**
Flower richness × Pollinator guild	9.64	2	**0.008**
(c) Specialization (*d*′)			
Flower richness	14.78	1	**0.0001**

*Notes*: The χ^2^, the degrees of freedom (df), and the *p* values were calculated based on likelihood ratio tests. Significant *p* values are shown in boldface type.

## DISCUSSION

In this study, we showed that resource diversity at local and landscape scales were significant predictors of the abundance and richness of wild pollinators, the structure of their pollination interactions and their diet breadth and specialization. Resource‐diverse habitats held richer and more abundant wild pollinator communities. Flower richness and landscape heterogeneity led to more specialized and modular networks, and more functionally complementary pollinators than expected at random. In flower‐rich habitats neutral processes were less common, and wild pollinators widened their taxonomic and functional diets, but also increased their specialization in the networks (i.e., reduced the number of plant species they visited from those available).

### Wild pollinator abundance and richness

As expected, we found higher pollinator abundance in flower‐rich communities (Kennedy et al., 2013; Lázaro, Tscheulin, Devalez, Nakas, Stefanaki, et al., 2016), and in heterogeneous landscapes (Kennedy et al., 2013; Mallinger et al., 2016), although this last relationship was only marginally significant. We also expected wild pollinator richness to increase with flower richness (Gómez‐Martínez et al., 2020; Lázaro, Tscheulin, Devalez, Nakas, Stefanaki, et al., 2016) and with landscape heterogeneity (Andersson et al., 2013; Mallinger et al., 2016; Steckel et al., 2014), but we failed to find the latter relationship. Heterogeneous landscapes may present a high variety of different habitats and microhabitats (Holzschuh et al., 2008), which increase landscape complementation (Brotons et al., 2004; Fahrig, 2003), and potentially harbor functionally different pollinators (Lázaro & Alomar, 2019) that may need resources scattered in different habitats, such as nesting sites or feeding resources (Gathmann & Tscharntke, 2002; Klein et al., 2004). Therefore, while local flower richness positively influences both richness and abundance, our results seem to indicate that heterogeneous landscapes primarily enhance wild pollinator abundance, perhaps through the increase in nesting resources (Moreira et al., 2015). Interestingly, agreeing with other authors (Lázaro et al., 2021; Ropars et al., 2020; Valido et al., 2019), we also found a negative relationship between honeybee abundance and wild pollinator richness. Honeybees are social insects and highly generalist feeders (Michener, 2007), with a great ability to communicate location of flower resources among colony members to respond quickly to resources availability (Beekman & Ratnieks, 2000). For these reasons, when honeybees are introduced at high abundances, they may out‐compete and displace wild pollinators by dominating the most abundant food resources (Hung et al., 2019). The displacement of wild pollinators by honeybees is a major concern because honeybees cannot replace the pollination services provided by wild species (Garibaldi et al., 2013). Therefore, the massive introduction of honeybees might deeply affect pollination interactions in natural ecosystems (Lázaro et al., 2021; Magrach et al., 2017) and agroecosystems, where it may drive to substantial economic losses (Cusser et al., 2021).

Beetles and wild bees were the guilds that responded more strongly to flower richness in terms of species richness, while richness of flies, wasps, and butterflies did not vary significantly along the flower richness gradient. Little is known about the effects of plant richness on beetle communities, and the findings are so far inconclusive, with some authors describing positive effects of plant diversity on beetle diversity (Woodcock et al., 2005), while others failing to find any relationship (Sjodin et al., 2008). Likely, the pattern found for beetles is related to the fact that is one of the most species‐rich guilds in our study system and by far the most abundant. On the other hand, positive effects of flower diversity on wild bee richness are largely described in literature (e.g., McCullough et al., 2021; Williams et al., 2015). It is not surprising that wild bee richness is closely related to flower richness, as these pollinators depend on flowering resources in all their life stages and castes (Michener, 2007). On the contrary, flies are a diverse group with many different feeding strategies in adult and larvae stages (Yeates & Wiegmann, 2005), which could be the reason for not finding a relationship between flower richness and fly richness in our system. Several studies have found positive effects of flower richness on hoverflies and/or bee flies (Lázaro, Tscheulin, Devalez, Nakas, & Petanidou, 2016; Meyer et al., 2009; Robertson et al., 2020), the main groups within flies specialized in flower feeding at the adult stage (e.g., Kastinger & Weber, 2001; Larson et al., 2001). In our communities, hoverflies and bee flies were scarce and the guild of flies was mostly composed by other types of flies (mainly Calliphoridae, Rhiniidae, Anthomyiidae, Empididae, and Chloropidae); therefore, flower‐specialist flies were pooled with the rest of flies for analyses (separating them in the analyses did not change the results; see Appendix S1: Table S13 and Figure S3). For this heterogeneous group, other factors not included in this study might be influencing their richness; for instance, aphidophagous species are found to thrive in agroecosystems, while saproxylic species are linked to forest (Jauker et al., 2009).

### The structure of plant–pollinator networks

Network specialization, modularity, and functional complementarity were always higher than expected at random, which is not surprising as different constraints as phenology, trait‐mismatching or pollinator preferences may prevent from a completely random distribution of interactions (Vázquez et al., 2007). They also became much higher than expected at random as both flower richness and landscape heterogeneity increased. The three metrics pointed in the same direction: high resource diversity at both local and landscape scales drives to more specialized and modular networks, where wild pollinator species decrease their interspecific niche overlap by using different flowering resources. Pollinators may segregate niches when resources are diverse to reduce competition, select more rewarding flowers and/or increase their foraging efficiency (Fründ et al., 2013; Venjakob et al., 2016), which may in turn lead to a higher specialization and modularity in the networks (Lázaro, Tscheulin, Devalez, Nakas, Stefanaki, et al., 2016; Olesen et al., 2007). Such increased niche differentiation might have important functional consequences, because functional complementarity has been shown to be positively related to plant reproductive success (Magrach et al., 2019). Therefore, heterogeneous landscapes might favor overall pollination service through the increase in functional complementarity in the networks.

Although the three analyzed metrics were related to landscape heterogeneity, network specialization (*H*_2_′) and modularity were affected by the landscape at a larger scale (1 km) than functional complementarity (250 m). Likely, these differences are due to the nature of the metrics, as *H*_2_′ and modularity are network‐level properties (Blüthgen & Klein, 2011), while functional complementarity is a group‐level property (Devoto et al., 2012) regarding just one trophic level of the network (wild pollinators, in our case). The effect on functional complementarity of landscape heterogeneity at 250 m might indicate that the processes involving the relationships among pollinator species are modulated at local scales of resource distribution. However, further work is needed to evaluate whether the scale‐patterns found here also are found in other plant–pollinator communities.

### Neutral processes versus active selection of flowers as determinants of interaction patterns

As expected, we found that local flower richness enhances the proportion of actively selected interactions that occur more than expected based on the relative abundance of the plant species (Vázquez et al., 2007). This might be related to non‐random process as trait matching (Jordano et al., 2003; Santamaría & Rodríguez‐Gironés, 2007; Vázquez et al., 2009), but also to higher selection of preferred/more rewarding flowers (Cusser et al., 2019), or to higher niche segregation to reduce competition (Inouye, 1978; Jeavons et al., 2020; Pasquaretta et al., 2019; Wignall et al., 2020) when flower availability increases. The relationship between flower richness and the proportion of actively selected interactions is similar to the relationship between flower richness and modularity, as wild pollinators preferentially visiting certain plant species is part of the process involved in the modular structure of interactions. However, while all the guilds increased their proportion of actively selected interactions with flower richness, landscape heterogeneity at 250 m slightly decreased the proportion for wild bees and beetles, but not for flies. The reasons for this are not clear, but some authors have argued that structurally complex habitats make efficient searching difficult (Brose et al., 2005; Laliberté & Tylianakis, 2010) and reduce the frequency of interactions, while less structurally complex habitats can improve search efficiency and increase the proportion of potential interactions (Laliberté & Tylianakis, 2010; Tylianakis & Morris, 2017). Perhaps landscape heterogeneity, as a measure of landscape complexity (Chaplin‐Kramer et al., 2011), reduces the proportion of actively selected interactions by hindering encounter probability between wild pollinators and their preferred plants. As in the case of functional complementarity, actively selected interactions were related to landscape heterogeneity at the 250 m scale, agreeing with the idea that processes occurring within the pollinator trophic level were influenced by the landscape at a smaller scale than processes influencing the whole network level. In any case, our results clearly indicate that interaction patterns are context dependent, and that wild pollinators show high plasticity in their diet, which allow them to adapt to different situations (Cusser et al., 2019; Inouye, 1978; Jeavons et al., 2020).

It is important to note that, even for the guilds with a higher proportion of actively selected interactions, still over a 55% of the interactions could be explained as random encounters, as estimated avoided interactions represented less than a 5% of total interactions. This indicates that more than half of the interactions occurring in these plant–pollinator communities are driven by stochastic processes and modulated by relative abundances (Vázquez et al., 2007).

### Diet breadth and specialization

Overall, our results show a high foraging plasticity as response to variation in local flower richness. We found that wild pollinators widen their taxonomic and functional diet as flower richness increases in the communities, i.e., pollinators fed on more plant species and with a higher variety of traits. However, there was no effect of landscape heterogeneity on taxonomic or functional diet. Cusser et al. (2019) also showed the diet breadth of two bee species was locally constrained by flowering resources and did not respond to landscape modifications. However, they just studied two bee species, while we have shown the same patterns studying over 33 species belonging to very different pollinator guilds (17 beetles, eight flies, and eight wild bees, that were present in at least 10 study sites and with at least five visits per site). Our result was expected, because a higher diversity of food resources implies more opportunities to feed on different flowering plant species. Despite that there were no differences among pollinators on how taxonomic diet breadth increased with flower richness, functional diet breadth increased more strongly for beetles than for flies and wild bees. In concordance with this, we also showed that the increase in the proportion of actively selected interactions as flower richness increased was faster for wild bees and flies than for beetles. It seems that beetles are highly opportunistic and randomly interact with plant species, therefore increasing their functional diet breadth when more flowering species are available. Flies and wild bees, on the contrary, seem to always feed on a relatively constant diversity of traits. Interestingly, despite the higher taxonomic and functional diet in more flower‐rich communities, species' specialization in the networks (*d*′) also increased with flower richness, consistently for the three guilds. Similarly, Lázaro, Tscheulin, Devalez, Nakas, Stefanaki, et al. (2016) showed that the specialization (*d*′) of bees, beetles and flies decreased along a gradient of grazing intensity (i.e., as communities became flower poorer). Our results are also in concordance with those reported by Kelly and Elle (2021) on the solitary bee *Andrena angustitarsa*, since they showed that this dietary generalist species increased its specialization (*d*′) as the abundance of Apiaceae flowers increased. Overall, our results indicate that pollinators can adjust their diet, by including more species in it as flower richness increases, but also segregating niches and feeding on a narrower subset of plants from those available. Further studies might perform deeper sampling of each population diets to confirm these patterns.

## CONCLUSIONS

Assessing how the diversity of resources at the local and landscape scales shape pollinator communities and their pollination interactions is essential to understand the effects of anthropogenic changes on the pollination services. This study shows that resource diversity at both scales enhances abundance and richness of wild pollinators and the complexity of plant–pollinator networks. Pollinators' diet breadth is mostly related to resources at the local scale, with wild pollinators widening their diets but also increasing their specialization in networks as flower richness increases. Our study indicates that pollinators are able to adapt their diet to cope with resource homogenization at local and landscape scales, induced by global change drivers. However, resource homogenization might lead to poor, generalist, and more functionally redundant pollinator communities, where pollination interactions are mainly driven by neutral processes, potentially hindering the maintenance of quality pollination services.

## CONFLICT OF INTEREST

The authors declare no conflict of interest.

## Supporting information


Appendix S1

## Data Availability

Data (Gómez‐Martinez et al., 2022) are available in Dryad at https://doi.org/10.5061/dryad.tht76hf1c.
