# Supplementary material for: Pollinator richness, pollination networks, and diet adjustment along local and landscape gradients of resource diversity
Source: Ecol Appl. 2022 Jun 26;32(6):e2634. doi: 10.1002/eap.2634 (PMC9539497; doi:10.1002/eap.2634)
Supplement: Supplementary file 1 — Appendix S1 [file EAP-32-e2634-s001.pdf]

Supporting Information. Carmelo Gómez-Martínez, Miguel A. González-Estévez, Joana Cursach and Amparo Lázaro. Pollinator richness, pollination networks, and diet adjustment along local and landscape gradients of resource diversity. Ecological Applications

Appendix S1

Table S1. Study sites, their coordinates, diversity at the local (flower richness) and landscape (landscape heterogeneity) scales. Landscape heterogeneity was calculated in radii of 250 meters, 500 meters and 1 kilometer buffer zones surrounding the center of the communities.

Site	Coordinates lat/lon	Flower richness	Landscape heterogeneity		
			250 m	500 m	1 km
Aloe Vera Farm	39.72139; 3.17184	43	0.211	0.605	1.191
Can Refila	39.67623; 3.18374	38	0.623	0.750	0.964
Can Vola Vola	39.54628; 2.80985	40	0.626	0.661	0.717
Carrer Betlem	39.39380; 2.84640	23	0.682	0.854	1.671
Es Cabanells	39.68937; 3.23535	39	1.086	1.086	1.375
Es Capdella	39.58026; 2.46473	52	0.730	0.927	1.66
Esporles	39.67318; 2.57537	37	0.627	1.387	1.939
Monti-Sion	39.49841; 3.01927	30	0.668	1.371	1.709
Muro	39.75011; 3.05956	56	0.964	1.258	1.48
Puntiro	39.59606; 2.80267	43	1.056	1.105	1.299
Sa Canova	39.43977; 2.97525	38	0.011	0.791	1.493
S'Alzinar	39.70549; 3.31425	38	0.453	0.754	1.138
Sa Ritxola	39.65353; 2.99953	41	0.426	0.761	1.243
S'Heretat	39.68254; 3.42145	68	0.201	0.987	1.335
Son Baco	39.38035; 3.04068	41	0.484	0.948	1.46
Son Doblons	39.69357; 3.19826	39	0.861	1.219	1.552
Son Ginard	39.41604; 3.07892	64	0.258	0.637	1.166
Son Gual	39.57082; 2.83420	43	0.005	0.783	1.287
Son Mut Nou	39.46140; 2.82563	32	0.876	1.041	1.328
UIB	39.64347; 2.64119	54	1.133	1.532	2.011

Table S2. Sampling completeness for pollinator and plant species and their interactions at each study site. According to Hsieh et al. (2016), sampling completeness is the proportion of the total of specimens belonging to the species registered in the sample, and it was calculated by using the Chao 1 asymptotic richness estimator (Chao et al., 2014). Function used was iNEXT from r-package *iNEXT* ver. 2.0.20 (Hsieh et al., 2016). Sampling completeness for pollinator species and interactions was calculated by accumulating sampling rounds, while for plant species was calculated by accumulating 1x1 m sampling squares.

**Study sites**	**Sampling completeness (%)**		
	**Pollinator species**	**Plant species**	**Interactions**
Aloe Vera Farm	65.93	96.18	52.05
Can Refila	61.16	97.14	42.44
Can Vola Vola	56.71	95.46	36.78
Carrer Betlem	65.28	98.45	39.61
Es Cabanells	65.23	96.46	49.74
Es Capdella	61.93	95.86	34.38
Esporles	56.95	97.17	50.79
Monti-Sion	59.37	93.71	43.26
Muro	67.45	97.48	44.75
Puntiro	69.33	97.88	27.53
Sa Canova	68.49	94.62	41.25
S’Alzinar	54.37	92.72	32.57
Sa Ritxola	66.5	94.76	44.03
S’Heretat	71.57	94.42	41.01
Son Baco	54.01	95.21	32.66
Son Doblons	49.89	97.64	39.39
Son Ginard	74.83	95.09	47.47
Son Gual	63.78	96.74	44.32
Son Mut Nou	62.16	97.77	36.95
UIB	60.26	96.35	39.68

Table S3. Pollinator community at each study site. Wild pollinator richness (Obs) are the observed values; Wild pollinator richness (50% SC) are the estimated values at 50% of sampling completeness (Hsieh et al., 2016). Wild pollinator abundance and richness, and number of links correspond to total values recorded per site.

Study site	Wild pollinator abundance	Wild pollinator richness (Obs)	Wild pollinator richness (50% SC)	Honeybee abundance	Number of links
Aloe Vera Farm	500	56	38	6	89
Can Refila	469	47	35	59	69
Can Vola Vola	226	56	49	140	83
Carrer Betlem	804	72	50	58	109
Es Cabanells	537	50	36	139	95
Es Capdella	563	87	67	25	170
Esporles	440	42	35	128	60
Monti-Sion	314	45	38	63	61
Muro	868	88	66	10	183
Puntiro	420	58	45	19	109
Sa Canova	257	52	34	93	89
S'Alzinar	310	50	47	10	92
Sa Ritxola	571	71	53	32	115
S'Heretat	706	88	58	79	214
Son Baco	320	69	65	50	103
Son Doblons	342	40	47	134	53
Son Ginard	699	76	54	138	176
Son Gual	224	57	45	98	86
Son Mut Nou	344	65	52	15	102
UIB	721	68	52	74	140

Table S4. Wild pollinator richness by guild at each study site. Standardized values within parentheses. Standardization of richness by guild was carried out by bootstrapping of species from the standardized overall richness values (Gotelli & Colwell 2001). For each site, it was carried out a 100 randomized subsampling of species according to the standardized overall number of pollinator species, and the subsampled species were categorized in one of the visitor guilds for each subsampling. Presented values within parentheses are mean values of the 100 randomized values of richness for each guild.

Study site	Beetles	Butterflies	Flies	Wasps	Wild bees	Total
Aloe Vera Farm	28 (19)	3 (2)	14 (10)	4 (3)	7 (5)	56 (38)
Can Refila	14 (10)	1 (1)	21 (16)	2 (2)	9 (7)	47 (35)
Can Vola Vola	16 (14)	0 (0)	25 (22)	4 (4)	11 (10)	56 (49)
Carrer Betlem	21 (15)	6 (4)	28 (20)	5 (4)	12 (8)	72 (50)
Es Cabanells	18 (13)	3 (2)	13 (9)	6 (4)	10 (7)	50 (36)
Es Capdella	26 (20)	6 (4)	19 (15)	9 (7)	27 (21)	87 (67)
Esporles	12 (10)	1 (1)	13 (11)	3 (3)	13 (11)	42 (35)
Monti-Sion	16 (13)	3 (3)	18 (15)	2 (2)	6 (5)	45 (38)
Muro	32 (24)	7 (5)	22 (17)	4 (3)	23 (17)	88 (66)
Puntiro	20 (16)	5 (4)	20 (15)	2 (2)	11 (9)	58 (45)
Sa Canova	16 (11)	3 (2)	19 (12)	2 (1)	12 (8)	52 (34)
S’Alzinar	22 (21)	2 (2)	13 (12)	3 (3)	10 (9)	50 (47)
Sa Ritxola	28 (21)	0 (0)	29 (22)	3 (2)	11 (8)	71 (53)
S’Heretat	33 (22)	5 (3)	25 (16)	3 (2)	22 (14)	88 (58)
Son Baco	21 (20)	1 (1)	29 (27)	5 (5)	13 (12)	69 (65)
Son Doblons	17 (17)	0 (0)	14 (14)	2 (2)	7 (7)	40 (40)
Son Ginard	26 (18)	5 (3)	23 (17)	5 (3)	17 (12)	76 (54)
Son Gual	18 (14)	4 (3)	15 (12)	5 (4)	15 (12)	57 (45)
Son Mut Nou	20 (16)	2 (1)	27 (22)	8 (6)	8 (6)	65 (52)
UIB	26 (20)	1 (1)	13 (10)	9 (7)	19 (15)	68 (52)

Table S5. Results of the GLM showing the relationships between flower richness and landscape heterogeneity and wild pollinator network metrics (i.e., metrics when honeybees were excluded from the networks): (A) Network specialization (H_2'); (B) Modularity and (C) Functional complementarity. The χ^2, the degrees of freedom (*df*) and the *p* values are calculated based on Likelihood Ratio Tests. Significant *p* values are marked in bold.

**Model**	**Predictor**	χ^2	***df***	***p***
A) Network specialization (H_2')	Flower richness	8.09	1	**0.004**
	Landscape heterogeneity (1 km)	6.95	1	**0.008**
B) Modularity	Flower richness	13.83	1	**0.0002**
	Landscape heterogeneity (1 km)	4.80	1	**0.28**
C) Functional Complementarity	Flower richness	3.78	1	0.052
	Landscape heterogeneity (250 m)	3.11	1	0.078

Table S6. Correlations among the study network metrics and other metrics typically calculated in literature. Non-italic values are Pearson's correlation coefficients (up-right corner); Italic values are *p* values of the correlations (bottom-left corner). Significant correlations are marked in bold.

	H_2'	Modularity	Functional Complementarity	wNODF	Interaction Diversity
H_2'		0.244	0.009	-0.316	**-0.554**
**Modularity**	*0.301*		0.152	**-0.816**	0.432
**Functional Complementarity**	*0.968*	*0.524*		-0.210	0.054
**wNODF**	*0.175*	**< *0.0001***	*0.374*		-0.318
**Interaction diversity**	***0.011***	*0.057*	*0.823*	*0.172*	

Table S7. Functional traits of the plant species in the study sites. The traits used, its functional attributed and the source where it was collected are given. For bicolored flowers, flower color was defined by the predominant color of the perianth.

**Trait**	**Functional attributes**	**Reference**
Type of floral unit	apetalous, flower, pseudanthium	Field observations
Floral unit size	0 (mm), <5, 5-10, >10	Castroviejo S. (coord. gen.). Flora iberica 1–8, 10–15, 17–18, 21. Madrid: Real Jardín Botánico, CSIC; 1986–2012.
Floral asymmetry	actinomorphy, zygomorphy	Field observations
Flower color	blue, green, pink, purple, red, white, yellow	Field observations
Flowering onset	Number of days since beginning of the year	Field observations
Flowering length	Number of days from onset to end	Field observations

Table S8. Pollinator species included in the analyses of the breadth. The species were present in at least 10 study sites and with at least 5 visits.

Species	
Beetles	Flies
Anaspis sp.	*Delia platura*
Anthaxia dimidiata	*Empis genualis*
Attalus lusitanicus	*Empis tessellata*
Bruchidius seminarius	*Episyrphus balteatus*
Clanoptilus abdominalis	*Oscinimorpha longirostris*
Dasytes nigroaeneus	*Pollenia sp.*
Dasytes terminalis	*Sarcophaga sp.*
Dasytes tristiculus	*Stomorhina lunata*
Isomira sp.	Wild bees
Mordellistena sp.	*Andrena fabrella*
Oedemera barbara	*Andrena flavipes*
Oedemera flavipes	*Bombus terrestris*
Oxythyrea funesta	*Eucera oraniensis*
Psilothrix aureola	*Halictus gemmeus*
Psilothrix viridicoerulea	*Halictus scabiosae*
Stenopterus ater	*Lasioglossum malachurum*
Tropinota squalida	*Lasioglossum villosulum*

Table S9. Akaike Information Criterion corrected for minimum sample sizes (AICc, Calcagno & de Mazancourt 2010) for best (italics) and alternative model (ΔAIC < 2) of wild pollinator abundance. Also shown are *p* values of the variables in the best and the alternative model (significant in bold)

**Response variable**	**Best and Alternative models**	***p***	**AICc**
a) Wild pollinator abundance	*Flower richness + Landscape heterogeneity (1 km)*	***0.016**; 0.062*	*269.4*
	Flower richness	**0.039**	269.5
	Landscape heterogeneity (1 km)	0.149	271.4

Table S10. Significance of network level metrics. Obs are the metric values from observed networks; Mean are the mean value of a 100 null models; lowerCI95 and upperCI95 are the lower and upper confidence limits of the 100 null models; Test shows if the observed value is significantly higher, lower or is within the CI; z score are the deviation of the observed value from the null expectation.

Network specialization (H_2')

**Study site**	**Obs**	**Mean**	**lowerCI95**	**upperCI95**	**Test**	**z score**
Aloe Vera	0.516	0.113	0.110	0.115	Higher	26.617
Can Refila	0.630	0.096	0.093	0.099	Higher	28.023
Can Vola Vola	0.557	0.109	0.106	0.111	Higher	30.353
Carrer Betlem	0.632	0.065	0.063	0.067	Higher	48.599
Es Cabanells	0.399	0.086	0.084	0.088	Higher	25.541
Es Capdellà	0.580	0.134	0.132	0.136	Higher	34.233
Esporles	0.659	0.062	0.060	0.064	Higher	46.886
Monti-Sion	0.595	0.120	0.117	0.123	Higher	26.943
Muro	0.534	0.117	0.115	0.118	Higher	45.325
Puntiró	0.585	0.130	0.128	0.132	Higher	38.095
Sa Canova	0.549	0.143	0.140	0.146	Higher	20.680
S’Alzinar	0.547	0.139	0.137	0.141	Higher	29.905
Sa Ritxola	0.615	0.130	0.128	0.132	Higher	35.572
S’Heretat	0.471	0.110	0.109	0.112	Higher	47.148
Son Baco	0.631	0.161	0.158	0.165	Higher	23.194
Son Doblons	0.819	0.082	0.080	0.084	Higher	56.424
Son Ginard	0.495	0.111	0.109	0.112	Higher	54.317
Son Gual	0.603	0.148	0.145	0.151	Higher	22.347
Son Mut Nou	0.449	0.160	0.157	0.163	Higher	16.622
UIB	0.594	0.086	0.084	0.087	Higher	71.198

Table S10. (Continuation)

Modularity						
**Study site**	**Obs**	**Mean**	**lowerCI95**	**upperCI95**	**Test**	**z score**
Aloe Vera	0.419	0.139	0.137	0.141	Higher	27.011
Can Refila	0.347	0.105	0.103	0.107	Higher	24.396
Can Vola Vola	0.511	0.169	0.167	0.171	Higher	30.987
Carrer Betlem	0.440	0.099	0.098	0.101	Higher	40.258
Es Cabanells	0.320	0.111	0.109	0.112	Higher	24.356
Es Capdellà	0.584	0.230	0.228	0.232	Higher	42.244
Esporles	0.454	0.103	0.101	0.105	Higher	30.857
Monti-Sion	0.404	0.136	0.134	0.138	Higher	24.210
Muro	0.504	0.166	0.165	0.167	Higher	51.994
Puntiró	0.538	0.191	0.189	0.193	Higher	32.495
Sa Canova	0.484	0.202	0.199	0.204	Higher	21.147
S'Alzinar	0.543	0.236	0.234	0.238	Higher	30.748
Sa Ritxola	0.471	0.160	0.159	0.162	Higher	37.144
S'Heretat	0.562	0.202	0.201	0.203	Higher	50.115
Son Baco	0.495	0.213	0.211	0.215	Higher	22.978
Son Doblons	0.528	0.120	0.118	0.121	Higher	42.385
Son Ginard	0.496	0.171	0.170	0.173	Higher	43.714
Son Gual	0.537	0.221	0.219	0.223	Higher	25.570
Son Mut Nou	0.413	0.209	0.207	0.211	Higher	19.620
UIB	0.608	0.152	0.151	0.154	Higher	57.964

Functional complementarity						
**Study site**	**Obs**	**Mean**	**lowerCI95**	**upperCI95**	**Test**	**z score**
Aloe Vera	313.094	210.013	208.638	211.387	Higher	12.337
Can Refila	325.756	183.478	182.034	184.921	Higher	16.215
Can Vola Vola	381.600	236.944	235.698	238.190	Higher	19.099
Carrer Betlem	767.008	575.828	573.508	578.148	Higher	13.553
Es Cabanells	489.534	315.024	312.623	317.425	Higher	11.957
Es Capdellà	474.235	234.220	231.723	236.717	Higher	15.813
Esporles	601.818	289.881	284.113	295.650	Higher	8.895
Monti-Sion	279.229	168.906	167.423	170.389	Higher	12.237
Muro	692.120	327.960	326.227	329.694	Higher	34.553
Puntiró	424.466	243.730	242.395	245.066	Higher	22.259
Sa Canova	284.360	189.216	187.420	191.012	Higher	8.714
S'Alzinar	286.375	152.704	150.899	154.509	Higher	12.181
Sa Ritxola	402.548	209.709	207.327	212.091	Higher	13.316
S'Heretat	471.784	261.810	259.392	264.227	Higher	14.285
Son Baco	202.369	131.646	130.478	132.815	Higher	9.955
Son Doblons	408.514	254.282	253.167	255.397	Higher	22.754
Son Ginard	612.015	295.328	293.576	297.080	Higher	29.734
Son Gual	258.505	185.304	184.059	186.549	Higher	9.672
Son Mut Nou	256.019	218.338	217.072	219.605	Higher	4.893
UIB	637.590	311.771	309.130	314.412	Higher	20.295

Table S11. Results of the model showing the relationships between flower richness and landscape heterogeneity and wild pollinator richness by guild, with hoverflies and bee flies as separated groups from the rest of the flies. The χ^2, the degrees of freedom (*df*) and the *p* values are calculated based on Likelihood Ratio Tests. Significant *p* values are marked in bold.

**Model**	**Predictor**	χ^2	***df***	***p***
Wild pollinator richness by guild	Flower richness	14.770	1	< **0.0001**
	Pollinator guild	29.059	6	< **0.0001**
	Honeybee abundance	7.462	1	**0.003**
	Flower richness × Pollinator guild	17.939	6	**0.004**

Fig. S1 Correlations among (A) the overall proportion of actively selected interactions (per study site) and raw values and z scores of functional complementarity; and (B) specialization (d') of each pollinator guild per study site and the proportion of actively selected interactions. For each relationship it is shown the Pearson's product-moment correlation (*r*) and the *p* value.

A

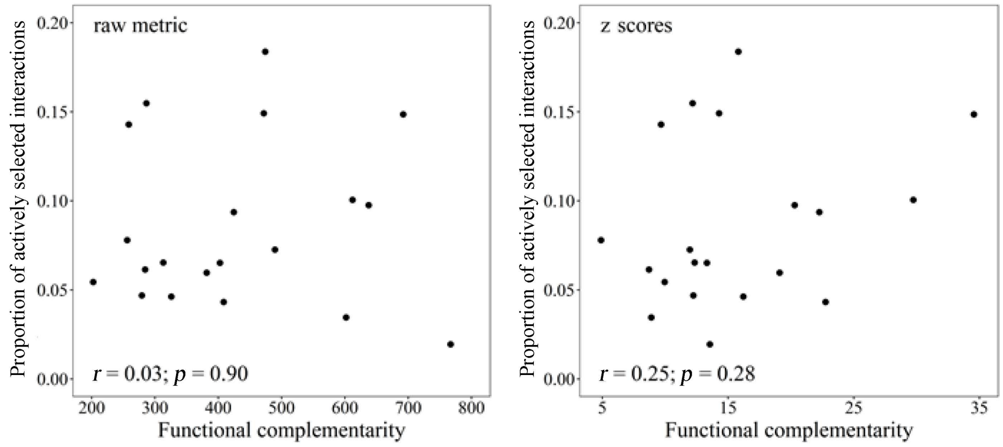

B

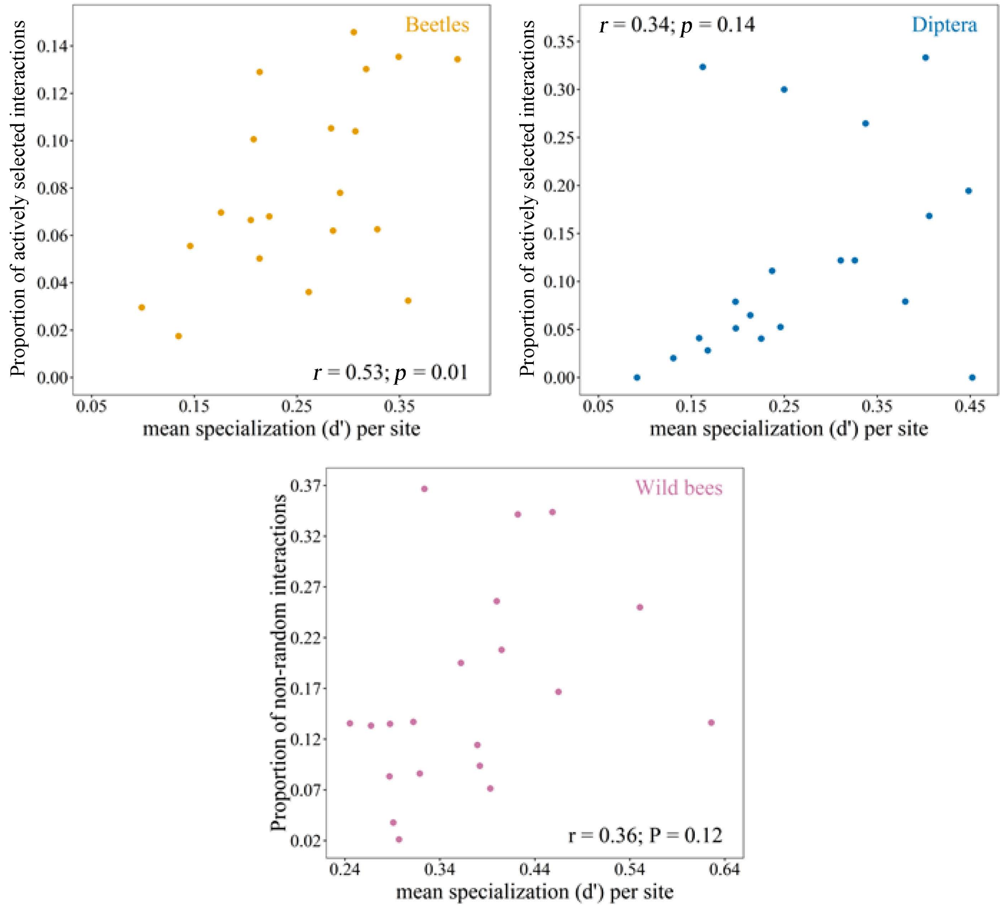

Fig. S2 Scatter plots for the relationships between landscape heterogeneity (landscape diversity) at different scales and flower richness (local diversity) or honeybee abundance; For each relationship it is shown the Pearson's product-moment correlation (*r*) and the *p* value.

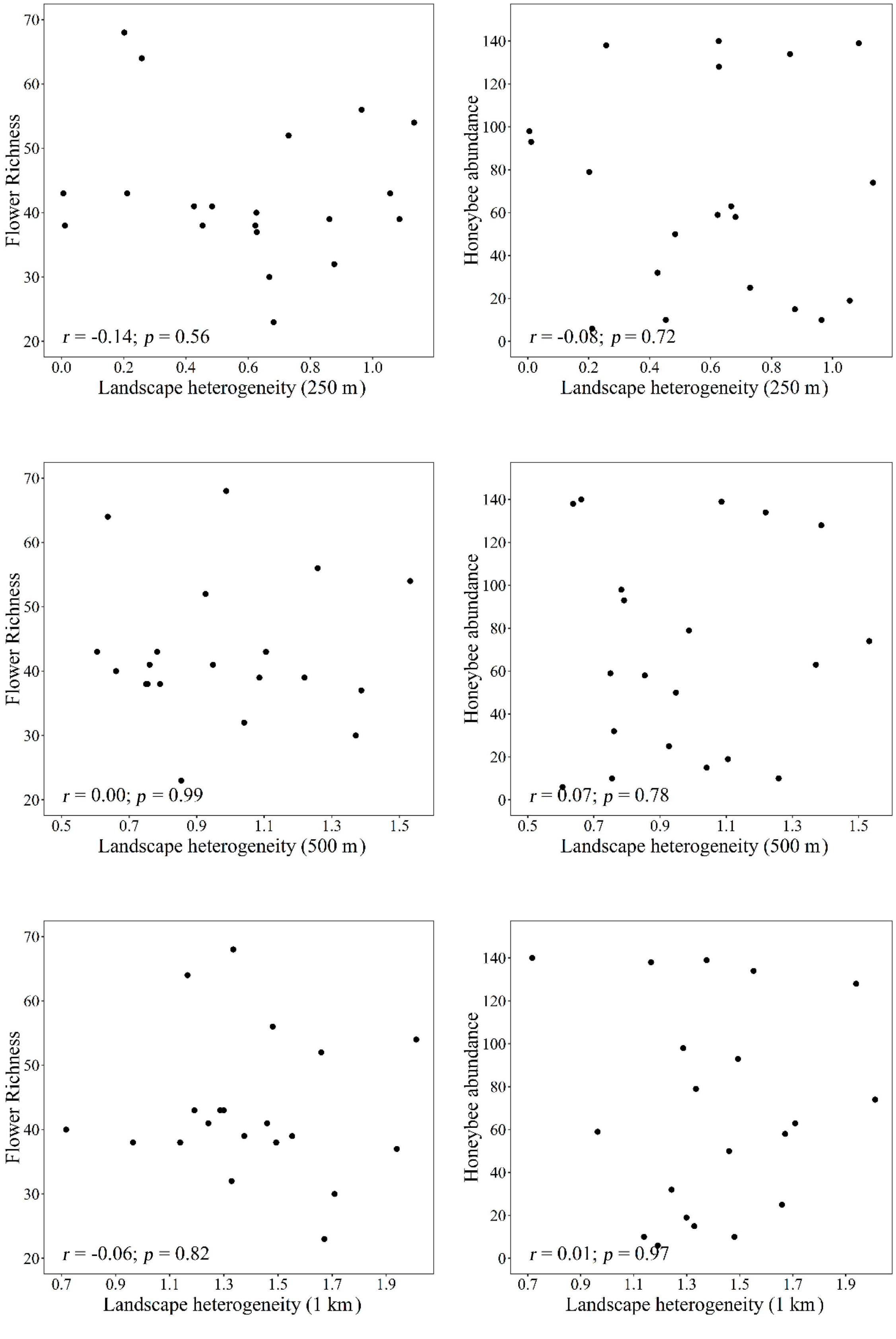

Fig. S3 Effect of local flower richness on wild pollinator richness by guild, with hoverflies and bee flies separated from the rest of the flies. The plot shows the relationship between local flower richness and the richness of the different wild pollinator guilds. Lines represent the estimates of the best model, the dots represent the data for each study site and guild, and the shaded area the confidence interval. Different letters indicate significant differences in mean (Estimated marginal means, lowercase) and trends (Estimated marginal means of linear trends, uppercase).

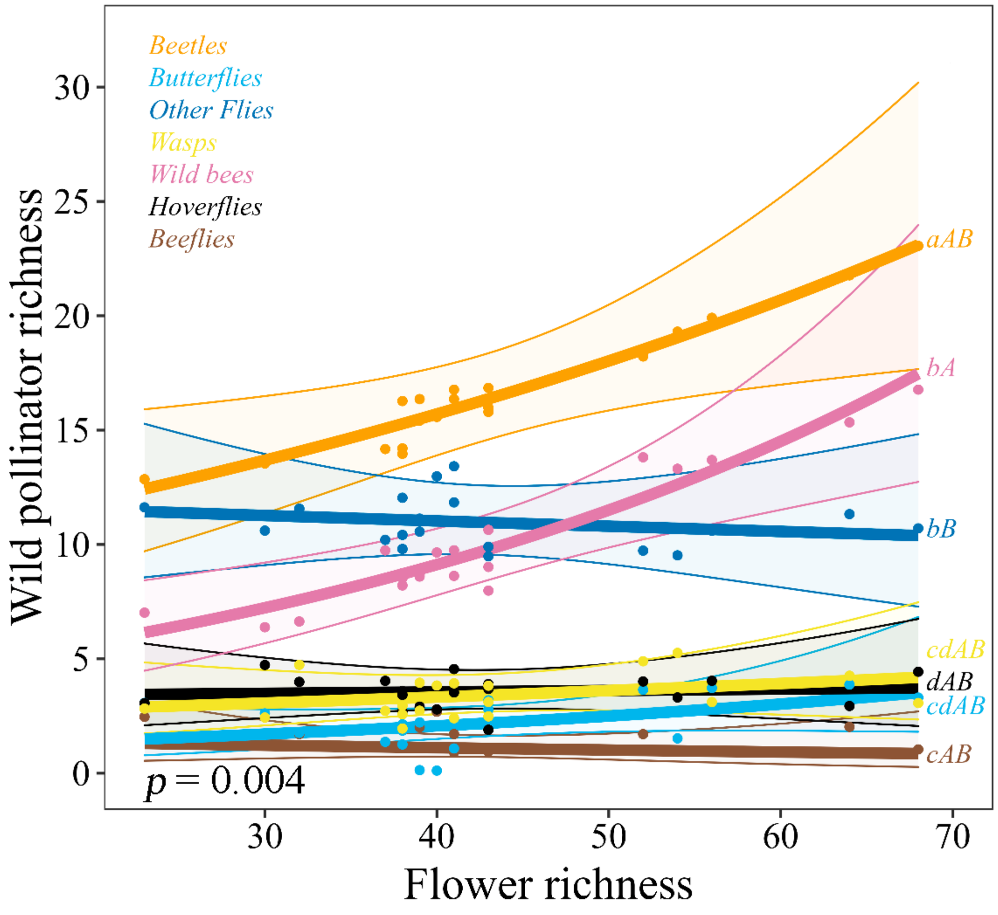

REFERENCES

Calcagno, V., and C. de Mazancourt. 2010. glmulti: An R Package for Easy Automated Model Selection with (Generalized) Linear Models. Journal of Statistical Software **34**:1-29.

Chao, A., N. J. Gotelli, T. Hsieh, E. L. Sander, K. Ma, R. K. Colwell, and A. M. Ellison. 2014. Rarefaction and extrapolation with Hill numbers: a framework for sampling and estimation in species diversity studies. Ecological monographs **84**:45-67.

Gotelli, N. J., and R. K. Colwell. 2001. Quantifying biodiversity: procedures and pitfalls in the measurement and comparison of species richness. Ecology Letters **4**:379-391.

Hsieh, T., K. Ma, and A. Chao. 2016. iNEXT: iNterpolation and EXTrapolation for species diversity. R package version **2**:1-18.
